# Peritoneal dialysis-related peritonitis caused by *Pseudomonas* species: Insight from a post-millennial case series

**DOI:** 10.1371/journal.pone.0196499

**Published:** 2018-05-10

**Authors:** Wanhong Lu, Bonnie Ching-Ha Kwan, Kai Ming Chow, Wing-Fai Pang, Chi Bon Leung, Philip Kam-To Li, Cheuk Chun Szeto

**Affiliations:** 1 Nephrology Department, First Affiliated Hospital, Xi’an Jiaotong University, Xi’an, Shaanxi, China; 2 Carol and Richard Yu Peritoneal Dialysis Research Centre, Department of Medicine & Therapeutics, Prince of Wales Hospital, The Chinese University of Hong Kong, Shatin, Hong Kong SAR, China; Universidade Estadual Paulista Julio de Mesquita Filho, BRAZIL

## Abstract

**Background:**

*Pseudomonas* peritonitis is a serious complication of peritoneal dialysis (PD). However, the clinical course of *Pseudomonas* peritonitis following the adoption of international guidelines remains unclear.

**Methods:**

We reviewed the clinical course and treatment response of 153 consecutive episodes of PD peritonitis caused by *Pseudomonas* species from 2001 to 2015.

**Results:**

*Pseudomonas* peritonitis accounted for 8.3% of all peritonitis episodes. The bacteria isolated were resistant to ceftazidime in 32 cases (20.9%), and to gentamycin in 18 cases (11.8%). In 20 episodes (13.1%), there was a concomitant exit site infection (ESI); in another 24 episodes (15.7%), there was a history of *Pseudomonas* ESI in the past. The overall primary response rate was 53.6%, and complete cure rate 42.4%. There was no significant difference in the complete cure rate between patients who treated with regimens of 3 and 2 antibiotics. Amongst 76 episodes (46.4%) that failed to respond to antibiotics by day 4, 37 had immediate catheter removal; the other 24 received salvage antibiotics, but only 6 achieved complete cure.

**Conclusions:**

Antibiotic resistance is common amongst *Pseudomonas* species causing peritonitis. Adoption of the treatment guideline leads to a reasonable complete cure rate of *Pseudomonas* peritonitis. Treatment with three antibiotics is not superior than the conventional two antibiotics regimen. When there is no clinical response after 4 days of antibiotic treatment, early catheter removal should be preferred over an attempt of salvage antibiotic therapy.

## Introduction

Peritonitis remains the Archilles heel of peritoneal dialysis (PD) and is the major cause of technique failure [1,2]. Peritonitis caused by *Pseudomonas* species is a particularly common and serious complication in the Asia-Pacific region, and is often associated with a poor response to antibiotics as well as a high rate of catheter removal [3,4]. The 2016 update of the International Society of Peritoneal Dialysis (ISPD) guidelines for management of PD-related preritonitis recommends the use of dual antibiotic therapy with different mechanisms of action and treatment for 3 weeks [5]. Catheter removal is generally necessary if concomitant catheter infection is present [5].

There are several clinical studies that describe the clinical course of *Pseudomonas* peritonitis in substantial detail [3,4,6]. However, most of these reports were published over 10 years ago. In this study, we aim to understand the clinical course and treatment response of *Pseudomonas* peritonitis following the widespread dissemination of the ISPD guidelines [5,7–9] and standardization of clinical practice [10,11].

## Patients and methods

### Case selection

The study was approved by the Clinical Research Ethics Committee of the Chinese University of Hong Kong; all data were fully anonymized before access by the researchers. All procedures are in adherence to the Declaration of Helsinki. All episodes of PD peritonitis in our unit from 2001 to 2015 were reviewed. The diagnosis of peritonitis was based on at least two of the following criteria [5]: (1) Abdominal pain or cloudy peritoneal dialysis effluent (PDE), (2) leukocytosis in PDE (white blood cell count >100/μl), and (3) positive Gram stain or culture from PDE. Exit-site infection was diagnosed when there was purulent drainage, with or without erythema, from the exit site [12].

In the 15 year of study period, 2075 episodes of peritonitis were identified; 173 episodes (8.3%) were caused by *Pseudomonas* species. Twenty episodes were excluded from analysis because the PD effluent showed mixed bacterial growth. Of the remaining 153 episodes from 127 patients, their demographic characteristics, underlying medical conditions, previous peritonitis, recent antibiotic therapy, antibiotic regimen for the peritonitis episode, requirement of Tenckhoff catheter removal, and clinical outcome were reviewed.

### Patient management

Peritonitis episodes were treated with standard antibiotic protocol of our center, which was generally intra-peritoneal cefazolin and ceftazidime unless there was clinical contraindication. Antibiotic regimen for individual patient was modified when culture results were available. In general, patients received two effective anti-pseudomonal antibiotics for at least 21 days. In some patients, a third anti-pseudomonal antibiotic, usually a fluoroquinolone, was added on the discretion of individual nephrologist. If the PDE did not clear up after 5 days of effective antibiotics, either the Tenckhoff catheter was removed immediately or the antibiotics were changed (the “salvage antibiotics” group), as judged clinically by the individual nephrologist. The salvage antibiotic regimen was usually amikacin plus a beta lactam antibiotics (for example, cefepime, cefoperazone / sulbactam, piperacillin / tazobactam, imipenem / cilastatin, or meropenem). All patients received oral nystatin for prophylaxis of secondary fungal peritonitis.

When Tenckhoff catheters were removed, patients were put on temporary hemodialysis and the systemic antitibiotics were continued for at least 2 weeks. Tenckhoff catheter reinsertion was attempted in all cases at least 3 weeks after Tenckhoff catheter removal. As described previously, patients were switched to long-term hemodialysis only when catheter reinsertion failed [13].

### Clinical outcome

Primary response was defined as resolution of abdominal pain, clearing of dialysate, and PDE neutrophil count <100/μl on day 10 with antibiotics alone. Complete cure was defined as complete resolution of peritonitis by antibiotics alone without relapse or recurrence episodes within 4 weeks of completion of therapy.

All patients were followed for at least 6 months after their treatment was completed. Relapse peritonitis episode was defined as an episode that occurred within 4 weeks of completion of therapy of a previous episode with the same organism or being culture negative [5]. Repeat peritonitis episode was defined as an episode that occurred more than 4 weeks after completion of therapy of a previous episode with the same organism [5]. Patient mortality was defined as death from any cause during antibiotic treatment or death during temporary hemodialysis.

In addition, we also reviewed the change in peritoneal transport characteristics, dialysis adequacy and residual renal function before and after the episode of *Pseudomonas* peritonitis. The peritoneal equilibration test (PET) was performed 4 weeks after the episode of peritonitis, the dialysis adequacy and other parameters were evaluated as well. Their methods have been described in our previous studies [14,15].

### Statistical analysis

Statistical analysis was performed by SPSS for windows version 20.0 (IBM corporation, Armonk, NY). Results were expressed as frequencies and percentages for categorical variables, mean ± SD for continuous variables, and median and interquartile range for nonparametric data. Differences between groups were analyzed by Chi-square test and Wilcoxon rank sum test or unpaired and paired Student’s t as appropriate. A *P* value of < 0.05 was considered statistically significant. All probabilities were two-tailed.

## Results

**Fig 1:**
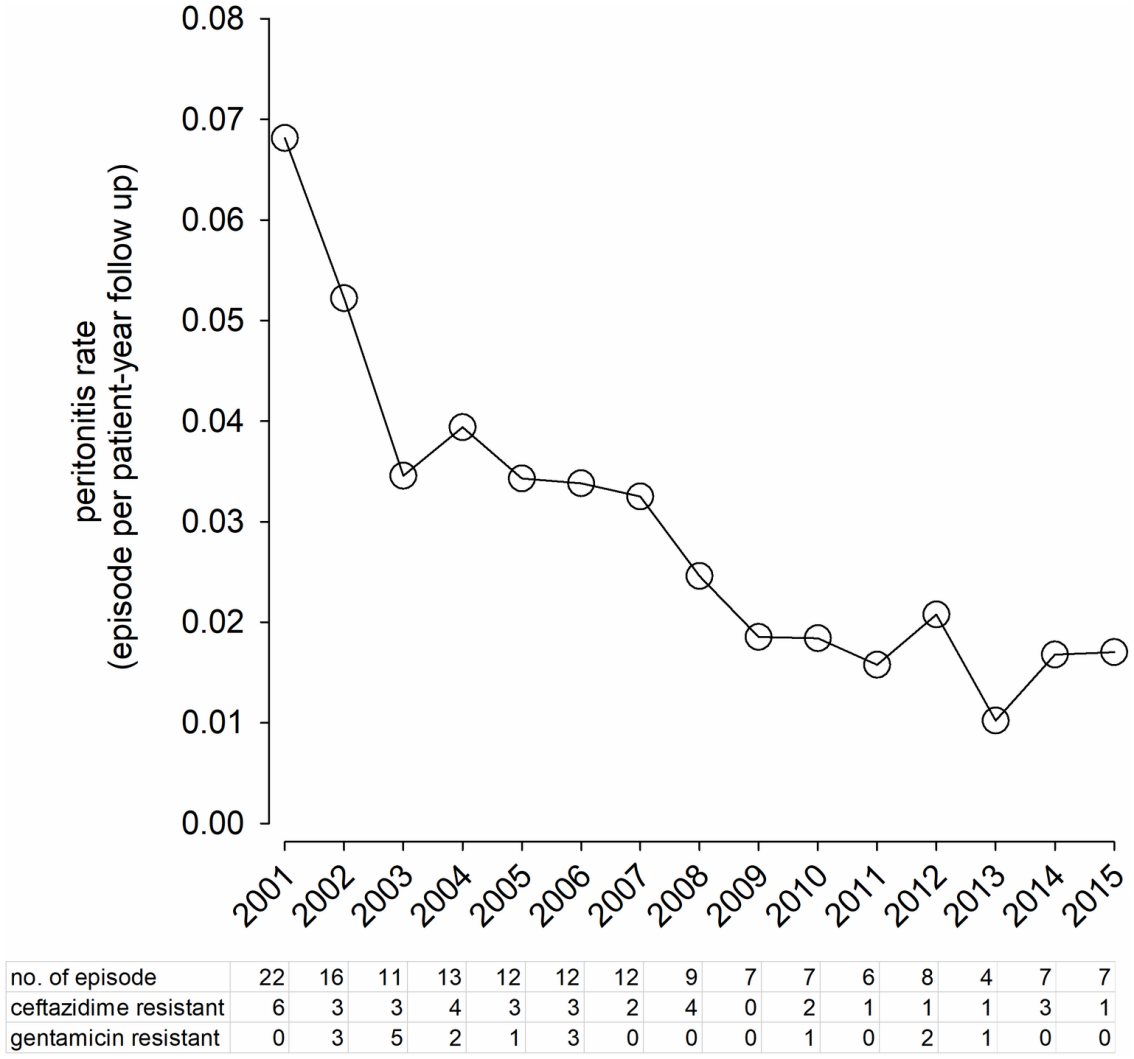
Change in incidence and evolution of antibiotic resistance of *Pseudomonas* peritonitis from 2001 to 2015.

All relevant data presented in this manuscript are available at S1 Table. From 2001 to 2015, 2075 episodes of peritonitis were identified in our unit; the overall peritonitis rate was 0.48 episode per patient-year. A total of 173 episodes (8.3%) were caused by *Pseudomonas* species; the absolute rate of *Pseudomonas* peritonitis was 0.04 episode per patient-year. The change in incidence of *Pseudomonas* peritonitis during the study period is summarized in Fig 1. In essence, there is a gradual reduction in the incidence of *Pseudomonas* peritonitis over the 15 years.

Twenty episodes were excluded from analysis because the PD effluent showed mixed bacterial growth. Of the remaining 153 episodes from 127 patients, their demographic and baseline clinical data are summarized in Table 1.

**Table 1:** Baseline characteristics of the patients*.

No. of patients	127
Gender (M:F)	50:77
Age (year)	57.8 ± 12.7
Duration of dialysis (month)	59.8 ± 45.2
Body mass index (kg/m^2^)	23.7 ± 4.4
Renal diagnosis, no. of patient (%)	
glomerulonephritis	37 (29.1%)
diabetic nephropathy	49 (38.6%)
hypertensive nephrosclerosis	5 (4.0%)
polycystic kidney	5 (4.0%)
urology / obstruction	4 (3.1%)
other	6 (4.7%)
unknown	21 (16.5%)
Major comorbidity, no. of patient (%)	
diabetes	56 (44.1%)
ischemic heart disease	35 (27.6%)
cerebrovascular disease	38 (29.9%)
peripheral vascular disease	10 (7.9%)
Charlson comorbidity score	6.1 ± 2.6
Type of PD, no. of patient (%)	
CAPD	119 (93.7%)
machine assisted PD	8 (6.3%)

*Abbreviations: PD, peritoneal dialysis; CAPD, continuous ambulatory peritoneal dialysis.

### Clinical and microbiological characteristics

The species of *Pseudomonas* isolated from PDE included *P*. *aeruginosa* (141 cases), *P*. *putida* (2 cases), *P*. *stutzeri* (1 case), and other unidentified species (9 cases). The bacteria were resistant to ceftazidime in 32 cases (20.9%), resistant to gentamicin in 18 cases (11.8%), and to both in 5 cases (3.3%). The evolution of antibiotic resistance over the 15 years is summarized in Fig 1. In spite of the decline in the incidence of *Pseudomonas* peritonitis, the proportion of bacterial isolate being antibiotic resistant remained static during the study period.

All patients had cloudy dialysis effluent at presentation. The median initial white cell count was 3780 (inter-quartile range 1600–7800) per μL. In 20 episodes (13.1%), there was a concomitant exit-site infection. *Pseudomonas* species was isolated in 4 episodes (2.6%). In 24 episodes (15.7%), there was a history of exit site infection caused by Pseudomonas species in the past, but the exit site was clean at the time of *Pseudomonas* peritonitis.

The antibiotic regimen is summarized in Table 2. Briefly, the first line empirical antibiotic for Gram-negative coverage was ceftazidime in 132 episodes (86.3%), gentamicin in 13 episodes (8.5%), and imipenem / cilastin in 8 episodes (5.2%). Overall speaking, the most common regimen was ceftazidime plus gentamicin, which was used in 128 episodes (83.7%). As described above, the bacterial isolate was resistant to ceftazidime in 37 episodes, and a different beta-lactam antibiotic was used when that information was available. Similarly, the bacterial isolated was resistant to gentamicin in 18 episodes, and the regimen was changed to amikacin. In 40 episodes (26.1%), oral ciprofloxacin was added as the third antibiotic according to the clinical decision of individual nephrologist.

**Table 2:** Summary of antibiotic therapy.

first line empirical Gram negative coverage	no. of case (%)	second anti-pseudomonal antibiotics	no. of case (%)
Ceftazidime	132 (86.3%)	gentamicin	128 (83.7%)
		amikacin	3 (2.0%)
		ciprofloxacin	1 (0.6%)
Gentamicin	13 (8.5%)	ceftazidime	2 (1.3%)
		imipenem / cilastin	5 (3.3%)
		ciprofloxacin sulperone	4 (2.6%) 2 (1.3%)
imipenem / cilastin	8 (5.2%)	gentamicin	7 (4.6%)
		amikacin	1 (0.6%)

### Clinical outcome

**Fig 2:**
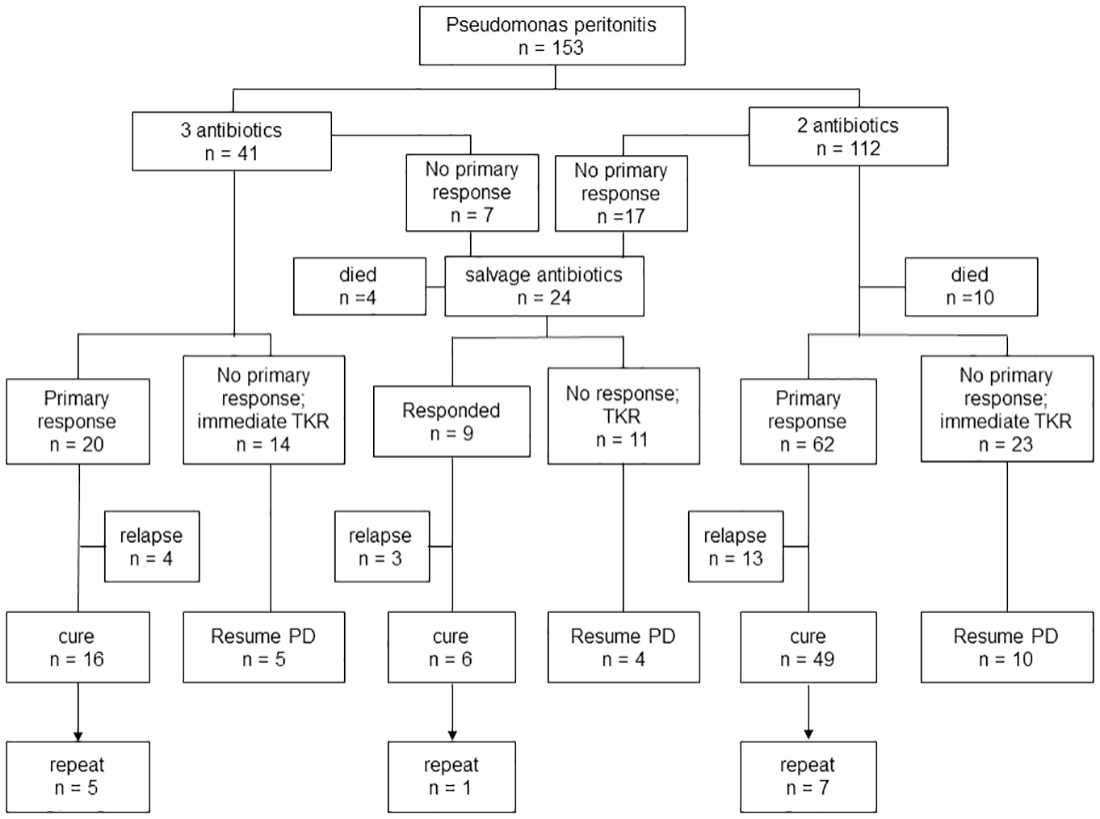
Summary of clinical outcome. (Abbreviations: TKR, Tenckhoff catheter removal; PD, peritoneal dialysis).

The clinical outcome is summarized in Fig 2. Fourteen patients (9.2%) died during the treatment of *Pseudomonas* peritonitis. The causes of death were peritonitis *per se* (9 cases), non-peritonitis infection (1 case), myocardial infarction (1 case), sudden cardiac arrest (1 case), stroke (1 case), and underlying malignancy (1 case).

The overall primary response rate was 53.6%; the complete cure rate was 46.4%. There was no difference in the primary response rate (53.8% vs 52.4%, p = 0.9) or complete cure rate (46.9% vs. 42.9%, p = 0.8) between episodes that received ceftazidime and other antibiotics as the initial antibiotic regimen. There was no difference in the complete cure rate between episodes treated with 3 and 2 antibiotics (47.06% vs 51.58%, p = 0.4), and the incidence of repeat peritonitis episode was marginally higher in the 3 antibiotics group (14.7% vs 7.37%, p = 0.177), but the difference was not statistically significant. The relapse rate had no difference between 3 and 2 antibiotics (11.8% vs 13.7%, p = 0.519). There were 9 out of 14 catheter removal cases in 3-antibiotics could not resume PD; the PD failure cases in 2-antibiotics group were 13 out of 23 cases. The comorbidities and baseline data were found no difference between 3 and 2 antibiotics groups before the episode of peritonitis (data not shown).

Seventy-one episodes (46.4%) showed no response by day 4. The catheter was immediately removed in 37 cases. In the other 24 episodes, salvage antibiotics therapy was used; complete cure was achieved in only 6 cases (25.0%), one-third of the cases relapsed, and there was 1 episode of repeat peritonitis. The chance of successfully resuming PD after catheter removal was higher in patients with immediate catheter removal than those with deferred removal for salvage antibiotics (40.5% vs 36.4%, p = 0.8) although the difference was not statistically significant. Demographic and baseline data between the catheter removal and the salvage antibiotic group were found mostly no difference before the episode of *Pseudomonas* peritonitis, except for gender and serum albumin level. The salvage antibiotic group had a lower serum albumin level than the catheter removal group (31.61 ± 6.81 vs 33.53 ± 4.42, p = 0.037), and prone to be male as well (p = 0.003, other data not shown).

### Effect of dialysis adequacy, nutritional status, and peritoneal transport

In 71 episodes, data on dialysis adequacy and nutritional status was available before and after the episode of *Pseudomonas* peritonitis, and their changes are summarized in Table 3. Briefly, there was a significant decline in residual glomerular filtration rate (GFR) and normalized protein nitrogen appearance, but no significant change in total Kt/V or other nutritional parameter after an episode of *Pseudomonas* peritonitis.

**Table 3:** Changes in peritoneal transport, nutritional status, and dialysis adequacy before and after the episode of *Pseudomonas* peritonitis*.

	before	after	*P* value**
dialysis adequacy			
total Kt/V	1.73 ± 0.33	1.76 ± 0.48	p = 0.9 ^a^
residual GFR (ml/min/1.73m^2^)	0.73 ± 1.36	0.68 ± 1.32	p < 0.0001 ^b^
urine output (L/day)	0.23 ± 0.37	0.23 ± 0.42	p = 0.8 ^a^
nutritional parameters			
hemoglobin (g/dL)	8.93 ± 1.83	8.87 ± 1.70	p = 0.7 ^a^
serum albumin (g/L)	32.4 ± 5.3	32.4 ± 4.7	p = 0.12 ^a^
NPNA (g/kg/day)	1.09 ± 0.24	1.06 ± 0.23	p = 0.005 ^a^
FEBM (%)	59.1 ± 13.8	55.9 ± 12.8	p = 0.3 ^a^
peritoneal transport			
D/P4	0.62 ± 0.16	0.66 ± 0.16	p = 0.4 ^a^
MTAC (ml/min/1.73m^2^)	8.32 ± 4.16	11.3 ± 7.77	p = 0.126 ^b^

*Abbreviations: GFR, glomerular filtration rate; NPNA, normalized protein nitrogen appearance; FEBM, fat-free edema-free body mass; D/P4, dialysate-to-plasma ratio of creatinine at 4 hours; MTAC, mass transfer area coefficient of creatinine.

**Data are compared by ^a^paired Student’s t test or ^b^Wilcoxon rank sum test.

In 20 episodes, PET was performed before and after the episode of *Pseudomonas* peritonitis, and their changes are summarized in Table 3. In essence, MTAC of creatinine and dialysate-to-plasma creatinine ratio at 4 hours (D/P4) increased after the episode of Pseudomonas peritonitis, although the increase did not reach statistical significance.

## Discussion

In this study, we reviewed 153 consecutive episodes of *Pseudomonas* peritonitis in our unit over 15 years. We find that dual antibiotics therapy is generally effective, and treatment with 3 antibiotics offers no obvious advantage. If the initial response to dual antibiotics treatment is not satisfactory, immediate catheter removal should be performed because the response to other salvage antibiotics is usually not rewarding. There are often deteriorations in residual renal function, nutritional status, and peritoneal ultrafiltration capability after *Pseudomonas* peritonitis, which may contribute to the long-term morbidity of the patient.

In this series, *Pseudomonas* species accounted for 8.3% of all peritonitis episodes, which is lower than 13.2% as reported in our previous study [4] but still higher than other reported series [3,16]. *Pseudomonas aeruginosa* accounted for over 90% of the isolates, which is considerably higher than other reports [16]. The higher incidence of *Pseudomonas* peritonitis and predominance of *P*. *aeruginosa* in Hong Kong may be due to unique local factors, such as the warm and humid climate that contributes to the colonization of *Pseudomonas* species [4]. It should be noted that amongst all *Pseudomonas* species, *P*. *aeruginosa* is the most invasive [17] and is associated with the need of catheter removal [16].

The incidence of antibiotic resistance is generally high in our patients, with over 20% isolates resistant to ceftazidime and over 10% to gentamicin. In contrast, our previous study between 1995 to 1999 reported the incidences of resistance being 4.8% in ceftazidime and 9.6% in aminoglycosides. Traditioinally, *P*. *aeruginosa* is prone to develop resistance in a variety of ways [18]. Liberal prescription of antibiotics was common among family physicians in Hong Kong [19], which possibly aggravate the problem.

Contrary to the rising incidence of antibiotic resistance, the incidence of concomitant exit site infection came down from over 45% in our previous report [4] to 13.1% in this study. The improvement is probably the result of our extra efforts in promoting exit site care and personal hygiene in our patients.

With strict adherence to the ISPD guidelines of treatment with two agents for 3 weeks, the primary response rate in this study was similar with our previous report [4], but the complete cure rate was substantially better (46.4% versus 22.1%). In this series, some episodes of *Pseudomonas* peritonitis were treated with 3-antibiotic. However, the relapse rates are almost identical between triple and double antibiotic regimens, and there was a trend of more repeat peritonitis episodes in the 3-antibiotic regimen, so did the PD failure rate. Given the retrospective nature of our study and despite the statistically similar baseline data in these two groups before the peritonitis, it is most possible that our observation represents a selection bias of choosing three antibiotics for those who were likely to have poor outcome. A randomized study is necessary to make further conclusion.

Because of practical difficulties (especially the availability of operating theatre), immediate Tenckhoff catheter removal was not possible and salvage antibiotics were used in 24 cases when PDE failed to clear up by day 5. The group that received salvage antibiotics, however, had a low response rate, and one third of them relapsed. Nonetheless, for the patients who failed to respond to salvage antibiotics and catheter removal was delayed, only 36.4% could return to PD later, which is not statistically different from patients with prompt catheter removal. Although we did not find any association between the timing of catheter removal and clinical outcome, we recommend prompt removal of catheter to avoid unnecessarily protracted course of antibiotics and prolonged peritoneal inflammation, which often leads to malnutrition and fluid accumulation.

In this study, we find that residual GFR and nutritional status worsen substantially after a single episode of *Pseudomonas* peritonitis. Moreover, the peritoneal permeability tends to switch to a higher transport rate as for the increasing trend of D/P4 and MTAC. Our result is consistent with previous studies, which showed that the number of peritonitis episode is an independent predictor of progression to anuria [20]. The worsening of ultrafiltration and the subsequent fluid overload may contribute to the long-term cardiovascular risk and a delayed impact on patient outcome.

In summary, we find that the incidence of antibiotic resistance is high amongst *Pseudomonas* species causing peritonitis. Nonetheless, adherence to the current treatment guideline has improved the complete cure rate of *Pseudomonas* peritonitis. Treatment with three antibiotics is not superior to the conventional two antibiotics regimen. When there is no clinical response after 4 days of antibiotic treatment, early catheter removal leads to a better outcome than salvage antibiotic regimens.

## Supporting information

S1 Table Dataset of the study. (XLSX)
